# The TERT promoter mutation status and MGMT promoter methylation status, combined with dichotomized MRI‐derived and clinical features, predict adult primary glioblastoma survival

**DOI:** 10.1002/cam4.1666

**Published:** 2018-07-09

**Authors:** Chang Shu, Qiong Wang, Xiaoling Yan, Jinhuan Wang

**Affiliations:** ^1^ School of Medicine Nankai University Tianjin China; ^2^ Tianjin Cerebral Vascular and Neural Degenerative Disease Key Laboratory Tianjin Neurosurgery Institute Tianjin Huanhu Hospital Tianjin China; ^3^ Pathology Department Tianjin Huanhu Hospital Tianjin China

**Keywords:** MGMT, MRI, primary glioblastoma, survival, TERT

## Abstract

**Purpose:**

This study aimed to integrate the TERT promoter mutation status, MGMT promoter methylation status, MRI‐derived features, and clinical features into a survival analysis model to better understand adult primary glioblastoma prognosis‐related markers.

**Method:**

A total of 304 adult glioblastoma samples collected after surgical resection were selected for retrospective analysis, and Sanger sequencing was performed to detect IDH and TERT promoter mutations. The methylation of the MGMT promoter was analyzed by pyrosequencing, and MRI‐derived and clinical features were dichotomized into easily acquired variables. Random survival forest analysis, Kaplan‐Meier analysis, Cox proportional hazard regression, and LASSO regression were performed for the survival analysis, and ROC analysis and Pearson's chi‐squared test were employed for the correlation analysis.

**Results:**

Wild‐type IDH was present in 89.8% of the adult glioblastoma samples, and TERT promoter mutations and MGMT promoter methylation were observed in 66.42% and 38.49% of all adult primary glioblastomas, respectively. Age and MGMT promoter methylation were identified as independent prognostic biomarkers, and the TERT promoter mutation status and MGMT promoter methylation status, when combined with other tumor‐related factors, generated several different survival subgroups. None of the factors investigated in this study predicted the MGMT promoter status, and MRI‐detected necrosis was positively associated with TERT promoter mutations.

**Conclusion:**

MGMT promoter methylation and TERT promoter mutations, combined with MRI‐derived and clinical features, revealed different survival subgroups with distinct responses to current treatments, and this information increases the ability to predict the survival of adult primary glioblastoma patients. MRI‐detected necrosis often indicates the presence of TERT promoter mutations.

## 1 INTRODUCTION

Approximately 90% of adult glioblastomas (GBMs) present as de novo GBMs (primary GBMs) without antecedent history of a less‐malignant precursor lesion, and the remaining cases progress from WHO lower grade (WHO II‐III) diffuse gliomas (secondary GBMs).^1^, ^2^ Isocitrate dehydrogenase 1 and 2 (IDH1/2) mutations are recognized as definitive diagnostic molecular biomarkers of secondary GBM and are more prognostically valuable than the history of tumor onset.^3^ Correspondingly, wild‐type IDH1/2 genotypes are considered robust biomarkers of primary GBM (<5%).^4^, ^5^ Epigenetic alterations affecting the MGMT promoter methylation status and genetic mutations in the TERT promoter are considered molecular signatures of GBM,^6^ but few studies have investigated the prognostic significance of these molecular features in adult primary GBM. The diagnosis and monitoring of GBMs in neurosurgical practice are often accomplished through MRI visualization. MRI‐derived features can be used to assess the entire tumor without sampling error, but few studies have attempted to integrate molecular alterations, MRI‐derived features, and clinical features into a single survival analysis model. Such a model would more robustly predict the outcomes of adult primary GBM patients than models that consider each factor independently.

## 2 MATERIALS AND METHODS

### 2.1 Patients and tumor samples

This retrospective study was approved by the Ethics Committee and Institutional Review Board of Tianjin Huanhu Hospital. In accordance with the Declaration of Helsinki, written informed consent for the use of clinical information and tissues was obtained from the patients.

**Figure 1:**
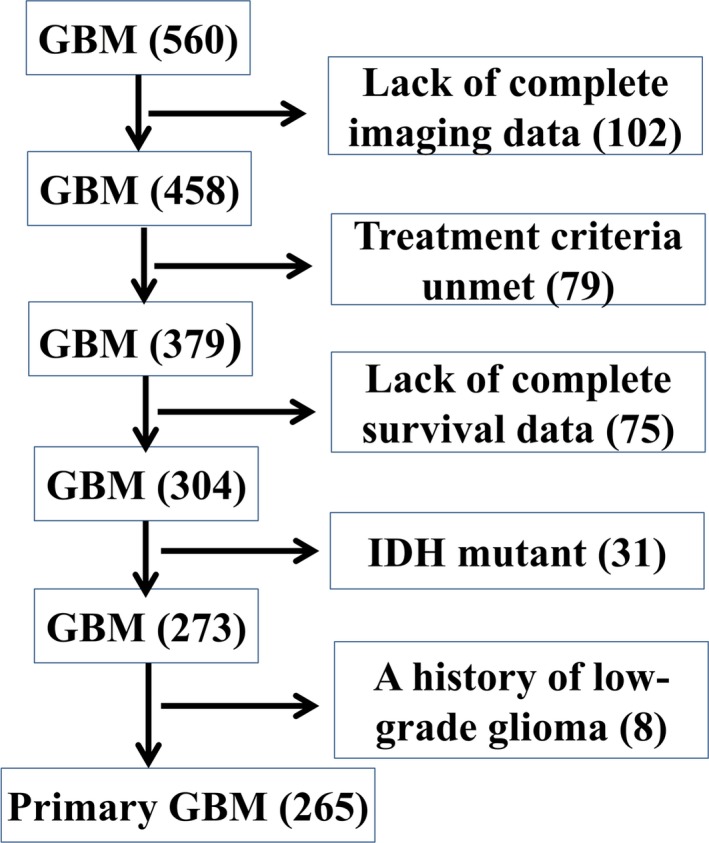
Flowchart of Patient Selection

A total of 560 adult GBM patients were analyzed in this retrospective study. These patients were preoperatively diagnosed as having a high‐grade glioma by neuroimaging (CT or MRI), and the glioma was confirmed as GBM by postoperative histopathology. Both the operations and the histopathological assessments were performed from January 2011 to January 2013. A diagram depicting the patient selection process is shown in Figure 1. We selected the included patients based on the stringent criteria defined by the CGCG clinical treatment guidelines.^7^ Briefly, the selected patients were treated with radiotherapy plus concomitant and adjuvant TMZ chemotherapy after maximal safe resection of the tumor. TMZ was administered at a daily dose of 75 mg/m^2^ (7 days a week) during radiotherapy.

### 2.2 DNA extraction and molecular analysis

The presence of hotspot mutations in IDH (IDH1‐R132, IDH2‐R140, IDH2‐R172) and TERT (C228T/C250T) in all the cases included in this study was detected by Sanger sequencing, as previously described.^8^, ^9^ The methylation status of the MGMT promoter was analyzed by pyrosequencing, as previously described.^10^

### 2.3 Analysis of MRI‐derived features

MRI sequences were acquired on a 1.5T scanner (GEMS 1234) and included SE T1WI (TR/TE: 2126/22), FRFSE T2WI (TR/TE: 4300/102), T2FLAIR (TR/TE: 8502/125) and diffusion‐weighted imaging sequences (TR/TE: 6000/76, *b* value: 0 s/mm^2^ and 1000 s/mm^2^). The parameters used for imaging were a section thickness of 6 mm, an intersection gap of 1 mm, a PFOV of 24 cm × 24 cm, and a 320 × 224 matrix. Contrast‐enhanced T1‐weighted images were acquired immediately following injection of the contrast agent Gd‐DTPA. Diffusion‐weighted imaging was performed prior to contrast‐enhanced T1‐weighted imaging. The ADC value was calculated as previously described.^11^ The MRI scans were interpreted by two neuroradiologists blinded to the patient outcome. We selected eight cardinal tumor‐related imaging features and dichotomized these features according to previous guidelines using the following criteria: ADC value,^12^, ^13^ peritumoral edema,^14^ contrast‐enhancing tumor (CET),^15^ necrosis,^16^ formation of cysts (cyst),^16^ noncontrast‐enhancing tumor (nCET),^17^ T1/Flair ratio,^16^ and deep white matter invasion.^15^ The exact dichotomy information is listed in Table 1.

**Table 1:** The relations between molecular biomarkers and adult primary GBM‐related features

Variables	MGMT promoter				TERT promoter				Ki‐67 LI			
	*M*	*U*	χ^2^	*P*	WT	Mut	χ^2^	*P*	*L*	*H*	χ^2^	*P*
ADC												
>ADC_mean	52	80	0.091	0.7633	47	85	0.482	0.4877	72	60	**6.3498**	**0.01174**
≤ADC_mean	50	83			42	91			52	81		
Edema												
Mild to moderate	47	56	3.629	0.0568	38	65	0.827	0.3632	44	59	1.1232	0.2891
Severe	55	107			51	111			80	82		
T1/Flair												
T1~Flair	44	62	0.680	0.4096	27	79	**5.214**	**0.0224**	56	50	2.587	0.1078
T1 < Flair	58	101			62	97			68	91		
CET												
Unobvious (<33%)	50	97	2.793	0.0946	44	103	1.975	0.1599	68	79	0.038	0.8458
Obvious (≥33%)	52	66			45	73			56	62		
Deep white matter invasion												
Absent	35	64	0.657	0.4176	34	65	0.041	0.84	47	52	0.0296	0.8635
Present	67	99			55	111			77	89		
Necrosis												
Unobvious (<33%)	47	61	1.947	0.163	46	62	**6.631**	**0.0100**	46	62	1.292	0.2558
Obvious (≥33%)	55	102			43	114			78	79		
Cysts												
Present	43	53	2.525	0.1121	34	62	0.226	0.6342	36	60	**5.221**	**0.0223**
Absent	59	110			55	114			88	81		
KPS												
≥80	58	80	1.523	0.2172	45	93	0.123	0.7258	59	79	1.887	0.1696
<80	44	83			44	83			65	62		
Age												
<50	52	99	2.436	0.1186	57	94	2.728	0.099	74	77	0.6912	0.4058
≥50	50	64			32	82			50	64		
Gender												
Female	49	81	0.069	0.7933	42	88	0.187	0.6657	54	76	2.8293	0.0926
Male	53	82			47	88			70	65		
nCET												
Obvious (≥33%)	44	55	2.366	0.124	30	69	0.763	0.3823	52	47	2.086	0.1486
Unobvious (<33%)	58	108			59	107			72	94		
MGMT promoter												
Methylated					32	70	0.364	0.5464	40	62	3.8236	0.051
Unmethylated					57	106			84	79		
TERT promoter												
WT	32	57	0.364	0.5464					45	44	0.765	0.3819
Mut	70	106							79	97		
Ki‐67												
High	40	84	3.824	0.051	45	79	0.765	0.3819				
Low	62	79			44	97						

### 2.4 Immunohistochemical (IHC) analysis for Ki‐67

An IHC analysis was performed to obtain the labeling index (LI) for Ki‐67, and IHC staining of Ki‐67 was performed using a primary anti‐Ki‐67 antibody (1:100 dilution; catalogue no. ab8191; Abcam, Shanghai, China). The IHC protocols were previously described in detail.^18^ We dichotomized the Ki‐67 LIs by the mean values obtained for tumor cases.^18^

### 2.5 Statistical analysis

Clinical demographic and MRI‐derived features were compared in combination with the MGMT promoter methylation status, the TERT promoter mutation status and the Ki‐67 LI using the Pearson chi‐squared test (R version 3.3.2). Random survival forest analysis was performed using the “randomForestSRC” and “ggRandomForests” R packages (ntree = 10 000 and nsplit = 10). LASSO analysis was completed using the “glmnet” R package. The optimal shrinkage parameter (lambda) was selected by cross‐validation. Survival curves were generated using the Kaplan‐Meier method and visualized with the “survival” R package. The log‐rank test was used to compare two or more survival curves. A multivariate Cox regression model was applied to assess the effects of the ADC value, age, peritumoral edema, TERT promoter mutation status, and MGMT promoter status on the survival of adult primary GBM patients. This model was also applied to assess the impact of KPS, peritumoral edema, age, and Ki‐67 LI on the survival of primary GBM patients with an unmethylated MGMT promoter and TERT promoter mutations. Overall survival (OS) was defined as the time from the first operation to death or the date of the last follow‐up examination. Survival was last assessed in August 2017. The receiver operating characteristic (ROC) analysis was performed using the “pROC” R package and visualized with the “ggplot2″ R package. Unless stated otherwise, *P *≤* *0.05 was considered to indicate significance.

## 3 RESULTS

### 3.1 TERT/MGMT promoter status and Ki‐67 LI in adult primary GBM

Three hundred four selected GBM samples (collected from patients aged at least 18 years) were assessed by Sanger sequencing to identify IDH1/2 mutations. Wild‐type IDH was present in 89.8% (273/304) of the total GBM samples. Among 273 GBM patients with wild‐type IDH, eight were previously diagnosed with low‐grade gliomas, accounting for 2.93% (8/273) of all GBM patients with wild‐type IDH. Two hundred sixty‐five wild‐type IDH GBM patients without antecedent history of low‐grade glioma were diagnosed as having primary GBMs (gross‐total resection rate: 72.6%, subtotal resection rate: 27.4%, 176 patients completed the above‐mentioned course of chemoradiation). TERT promoter mutations were observed in 66.42% (176/265) of all primary GBMs. Of these patients, 75% (132/176) had a C228T mutation, and 26.14% had a C250T mutation. Only two patients with primary GBMs had both C228T and C250T mutations, and most mutations were heterozygous. Pyrosequencing confirmed that the MGMT promoter was methylated in 38.49% (102/265) of the patients with primary GBMs. The mean Ki‐67 LI was 28% (range: 10%‐70%). Relationships between these three molecular biomarkers and other clinical demographic and MRI‐derived features are summarized in Table 1. A representative example of the main results is shown in Figure S1.

### 3.2 Prediction and validation of survival‐related variables

**Figure 2:**
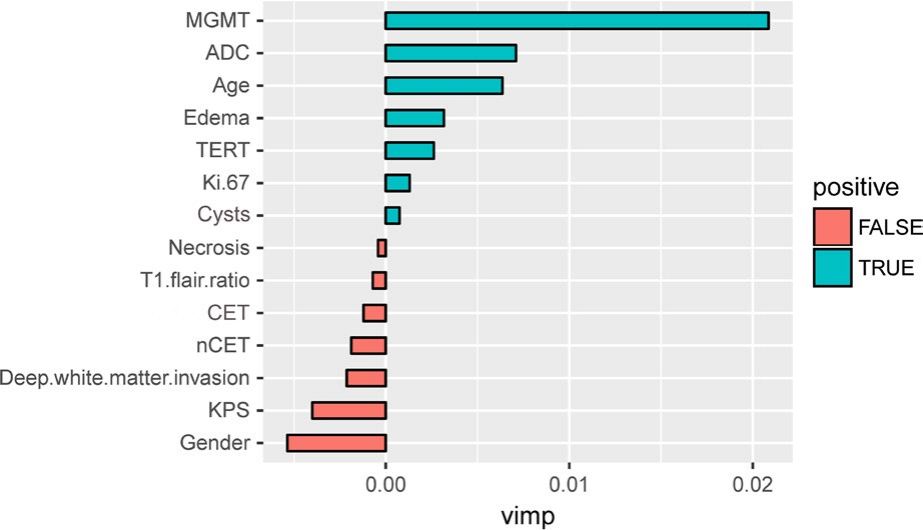
Top Five Variables (MGMT, ADC, Age, Edema, and TERT) Identified as Surrogate Survival‐Related Variables Through Variable Importance (vimp) Measurements According to a Random Survival Forest Analysis

**Figure 3:**
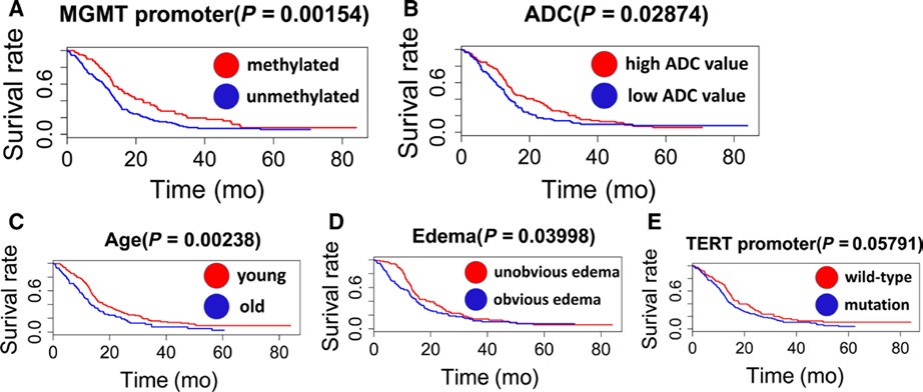
Kaplan‐Meier curves for the Five Key Variables (A‐E) Identified in the Random Survival Forest Analysis: (A) MGMT, (B) ADC, (C) Age, (D) Edema, and (E) TERT

A random survival forest analysis was performed to predict surrogate survival‐related variables, and five positive values were found to indicate the predictive power of the forest. The five key variables were MGMT promoter methylation status (MGMT), ADC value (ADC), age, tumor edema (edema), and TERT promoter mutation status (TERT) (Figure 2). The validation process indicates that the variables MGMT, ADC, age, and edema divided the primary GBM patients into two subgroups with significant differences in the predicted survival patterns (MGMT: *P *=* *0.00154, ADC: *P *=* *0.02874, age: *P *=* *0.00238, edema: *P *=* *0.03998) (Figure 3A‐D). TERT roughly divided the primary GBM patients into two different survival subgroups with a borderline‐adjusted significant *P* value (*P *=* *0.05791) (Figure 3E). A Cox PH regression model of the five variables was established, and the model exhibited a *P* value <0.001 in all three tests (likelihood ratio test, Wald test, and log‐rank test), indicating the significance of the Cox PH regression model and soundly rejecting the omnibus null hypothesis. In the Cox PH regression model, the covariates age and MGMT remained statistically significant (*P *<* *0.05) for OS, but the covariates ADC, edema, and TERT were not statistically significant (Table 2).

**Table 2:** Cox PH regression model for OS

Covariates	HR	95%CI	*z*‐score	*P*
ADC	1.184	0.8851‐1.646	1.138	0.255281
Age	1.710	1.2554‐2.329	3.403	0.000668
Edema	1.334	0.9946‐1.788	1.924	0.054366
TERT	1.287	0.9425‐1.758	1.587	0.112411
MGMT	1.788	1.3182‐2.427	3.734	0.000188

HR, hazard ratio; CI, confidence interval; *P*,* P* value

### 3.3 Subgroup analysis of the group with an unmethylated MGMT promoter and TERT promoter mutations

**Figure 4:**
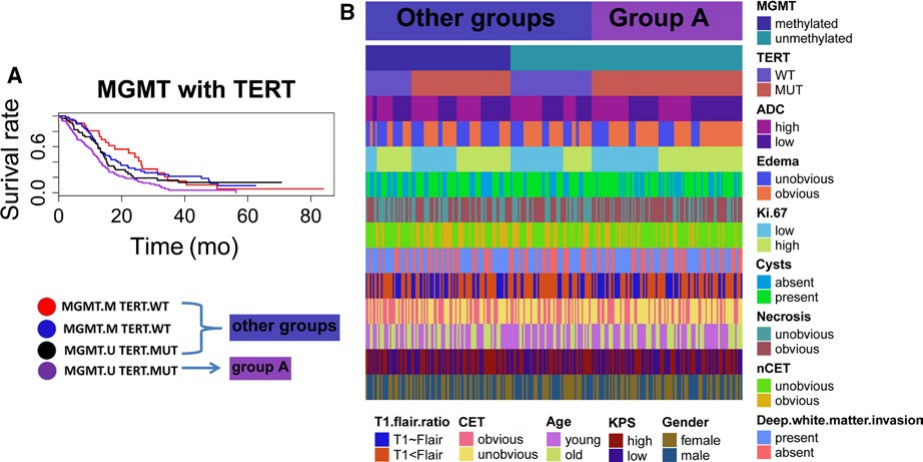
A, Analysis of the Survival of Patients with Different MGMT Promoter Methylation Statuses and TERT Promoter Mutation Statuses in the Research Cohort. B, Heatmap showing the distribution of adult primary GBM‐related factors in the subgroup with an unmethylated MGMT promoter and TERT promoter mutations (group A) and the other subgroups

Among the five important variables identified in the random survival forest analysis, the MGMT promoter methylation status and TERT promoter mutation status are key molecular tumor markers of GBM.^6^ We analyzed the relationship between the MGMT promoter methylation status and the survival benefit associated with the TERT promoter mutation status. The subgroup with both an unmethylated MGMT promoter and TERT promoter mutations represented 40% of all subgroups of different combinations of the MGMT promoter methylation status and TERT promoter mutation status (Table 1). A Kaplan‐Meier analysis showed that this subgroup demonstrated the worst OS among the subgroups (Figures 4A and S2A). This subgroup was examined further through a LASSO regression analysis, and four candidate prognostic‐related variables (age, edema, Ki‐67, and KPS) with nonzero coefficients in the LASSO analysis were identified through a cross‐matching test (Figure S2E‐G). The four covariates were introduced into the Cox PH regression model. Age and edema were identified as independent prognostic factors (Figure S2H). The heatmap shows the distribution of factors related to adult primary GBM in the subgroup with an unmethylated MGMT promoter and TERT promoter mutations and in the other subgroups (Figure 4B).

### 3.4 Analysis of multiple primary GBM‐related factors

**Figure 5:**
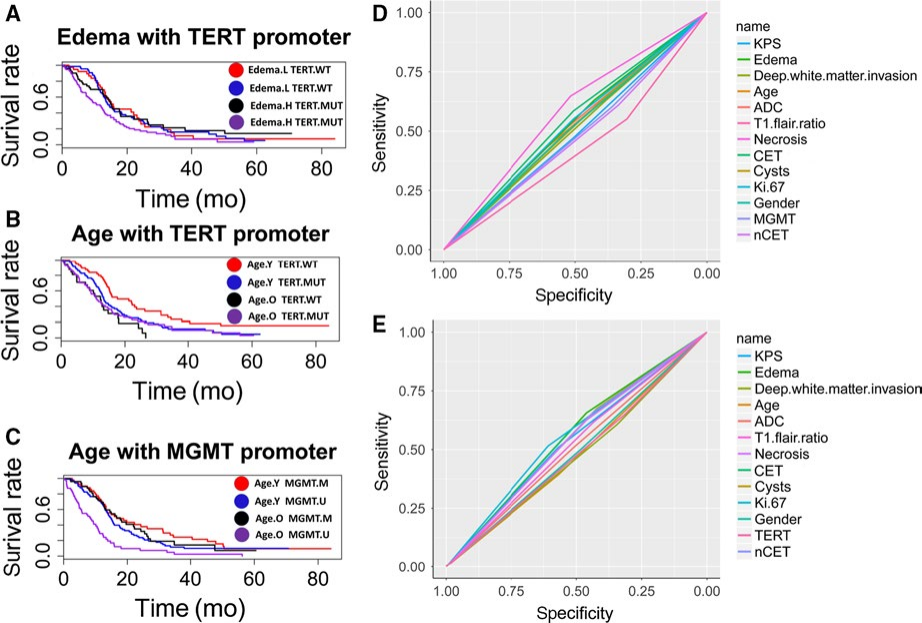
Kaplan‐Meier Curves Comparing Patients with Tumors with a TERT Promoter Mutation and Patients with Tumors with a Wild‐type TERT Promoter. The patients were stratified by the peritumoral edema level (A). Tumors bearing a TERT promoter mutation compared with wild‐type tumors (B) and tumors with a methylated MGMT promoter vs an unmethylated MGMT promoter (C). The tumors in (B) and (C) were stratified by age. ROC curves for TERT (D) and MGMT (E) promoter status among the adult primary GBM‐related factors

A multivariate survival analysis of the prognostic effect of the interaction among the five important variables identified in the random survival forest analysis (MGMT, TERT, age, edema, and ADC) was performed. A subgroup with obvious edema and TERT promoter mutations had an increasingly negative prognosis compared with the other subgroups with different edema and TERT promoter status combinations (Figures 5A and S2B). The subgroup combining younger age with a wild‐type TERT promoter exhibited enhanced OS compared with the other subgroups with different age and TERT promoter mutation status combinations (Figures 5B and S2C). The subgroup combining older age with an unmethylated MGMT promoter had poorer OS than the other subgroups with different age and MGMT promoter methylation status combinations (Figures 5C and S2D). We performed an ROC analysis to compare the sensitivities and specificities of the predictive ability of the TERT mutation status and the MGMT promoter methylation status among the other factors related to adult primary GBM listed in Table 1. The areas under the ROCs (AUCs) for these factors were determined and compared. As shown in Figure 5D, necrosis had the highest AUC value (0.5823) for predicting the TERT promoter mutation status. The AUC for necrosis was significantly different than those for KPS (AUC: 0.4886, *P *=* *0.03555), T1/Flair ratio (AUC: 0.4273, *P *=* *0.001013), CET (AUC: 0.5454, *P *=* *0.04617), gender (AUC: 0.514, *P *=* *0.04414), MGMT (AUC: 0.4809, *P *=* *0.019), and nCET (AUC: 0.4725, *P *=* *0.00712). As shown in Figure 5E, Ki‐67 had the highest AUC value (0.5616) for predicting the MGMT promoter methylation status. The AUC obtained for Ki‐67 was different from those for KPS (AUC: 0.5389, P = 0.02882), edema (AUC: 0.5586, *P *=* *0.009726), T1/Flair ratio (AUC: 0.5255, *P *=* *0.03345), necrosis (AUC: 0.5433, *P *=* *0.0218), cysts (AUC: 0.5482, *P *=* *0.01808), and nCET (AUC: 0.547, *P *=* *0.009551).

In addition, the TERT promoter mutation status was positively associated with the T1/Flair ratio (*P* = 0.0224) and necrosis (*P* = 0.01). The Ki‐67 LI was negatively associated with the ADC value (*P* = 0.01174) and cysts (*P* = 0.0223). No primary GBM‐related factors were associated with the MGMT promoter methylation status (Table 1).

## 4 DISCUSSION

Through a random survival forest analysis, we observed the following five key variables: MGMT promoter methylation status, TERT promoter mutation status, age, peritumoral edema and ADC value. The validation of their prognostic function by Kaplan‐Meier and Cox PH regression analyses revealed that age and the MGMT promoter methylation status were important prognostic markers that provide independent information. Primary GBM comprises 84.21% (265/304) of all adult GBM patients, and this ratio is lower than that reported in previous studies.^1^, ^6^ This discrepancy might be due to the classification of primary and secondary GBM based on the clinical history or IDH1/2 status in previous studies. However, we define primary GBM based on a combination of these two methods, and as a result, we ensured that the patients selected in our study are primary GBM patients and avoided the inclusion of secondary GBM patients, which could strongly interfere with the results. We did not find that the TERT promoter mutation status is an independent prognostic biomarker of primary GBM, even though the TERT mutation status analyses revealed borderline significance in the univariate survival analysis (Figure 3E). However, some GBM studies that included both primary and secondary GBM patients suggested that TERT promoter mutations are independently associated with a negative prognosis.^19^, ^20^ We speculate that the prognostic value of TERT promoter mutations in relation to poor survival is partly due to the IDH status. TERT promoter mutations without IDH mutations cannot reflect the different survival statuses of GBM patients.

Our results show that poor survival associated with TERT mutations was only observed in patients exhibiting high peritumoral edema, and increased survival associated with a wild‐type TERT promoter was only observed in young patients. We predicted that patients with high peritumoral edema that was preoperatively detected through MRI imaging and TERT promoter mutations that were postoperatively detected in tumor tissue would have a poorer prognosis and a higher mortality. These patients would likely need a more aggressive treatment strategy than young patients with a wild‐type TERT promoter, who may achieve a favorable oncologic outcome if treated. Another striking result of our study is that poor survival associated with an unmethylated MGMT promoter is detected only in aged patients who have undergone chemoradiotherapy after maximal safe resection of the tumor.

We also identified a primary GBM molecular subgroup of adult patients that showed promise for providing prognostic indications and differentiating treatment opinions. The subgroup with an unmethylated MGMT promoter and TERT promoter mutations exhibited worse survival than the other subgroups with different TERT promoter mutation status and MGMT promoter methylation status combinations (Figure 4A). TERT mutations might portend a more deleterious primary GBM if the MGMT promoter is unmethylated. Through a LASSO analysis, we discovered that the main factors that affected survival in this group were age and Ki‐67 positivity. The heatmap (Figure 4B) revealed that patients with low ADC values, obvious edema, a high Ki‐67 LI, obvious necrosis, unobvious nCET, deep white matter invasion, and obvious CET were enriched in this group. Some of these factors might represent MRI imaging markers for the subgroup of primary GBM patients with an unmethylated MGMT promoter and TERT promoter mutations that can be easily and noninvasively acquired. The exact mechanism that describes these results needs to be further explored and validated in future studies. We demonstrated that the Ki‐67 LI is negatively associated with the ADC value through Pearson's chi‐squared test (Table 1). A univariate survival analysis showed that a lower ADC value is associated with poorer survival in adult primary GBM patients (Figure 3B). Therefore, based on the heterogeneity of primary GBM, measuring the ADC value might help identify the most appropriate biopsy site.

The ROC analysis showed that Ki‐67 demonstrated the highest AUC value among primary GBM‐related factors for assessing the MGMT promoter methylation status. However, Pearson's chi‐squared test showed no statistical relationship between the Ki‐67 LI and MGMT promoter methylation status. No MRI‐derived features or clinical factors were found to predict the MGMT promoter methylation status. We speculate that the MGMT promoter methylation status might contribute to prognosis through mechanisms that differ from those of other primary GBM‐related factors and thus provides unique prognostic information for adult primary GBM. The ROC analysis demonstrated that necrosis had the highest AUC value for assessing the TERT promoter mutation status, and Pearson's chi‐squared test showed that necrosis was positively associated with the TERT promoter mutation status. Our study showed that TERT promoter mutations (66.42%) occur in many primary GBMs, and the frequency at which these were detected was in line with previous studies (58%‐75%).^21^, ^22^ TERT promoter mutations are frequency associated with malignant tumor progression and a capacity for enhanced cell proliferation.^23^ The unenhanced region on MRI, which represents pathological necrosis, reflects tumor progression. Tumor necrosis is caused by chronic ischemic injury due to rapid tumor proliferation.^24^ Our study confirms that the TERT promoter mutation status is correlated with pathological necrosis and that necrosis detected through MRI reflects the TERT promoter mutation status.

This study has several limitations. Our analysis was limited by its retrospective nature, and our findings were obtained from a single center. The results should be confirmed in a prospective, multicenter study. The sequencing method used in this study was not high‐throughput and could only report confirmed hotspot mutation sites. We cannot rule out the possibility of new mutation sites in the IDH and TERT promoters. Measurement of the ADC value can be affected by several technical factors, such as the timing of contrast agent administration, the coregistration of different images, and the selection of the region of interest (ROI) on the ADC map. The establishment of a uniform standard is necessary for the measurement of more accurate ADC values. Despite our attempts to minimize differences among treatments by only selecting patients who received standard surgery and concurrent chemoradiation therapy, as described in the CGCG clinical practice guidelines,^7^ the patients could have been exposed to a variety of other chemotherapies throughout their treatment. In addition, the surgeons were responsible for ensuring that the patients had undergone maximal safe resection, which can lead to selection bias and thereby affect the analysis of the results.

In summary, methylation of the MGMT promoter (38.49%) and mutations in the TERT promoter (66.42%) are prognostically important molecular events in adult primary GBM. The MGMT promoter methylation status and TERT promoter mutation status, combined with MRI‐derived and clinical features, can define different subgroups with distinct responses to the current treatment options. The combination of these factors results in a stronger survival prediction for adult patients with primary GBM. No imaging‐related features can predict the MGMT promoter methylation status. MRI‐detected necrosis is positively associated with the TERT promoter mutation status. If confirmed in prospective studies, these findings might have clinical implications in identifying critical factors for improving patient outcomes and treatment selection.

## CONFLICT OF INTEREST

The authors declare that no conflicts of interest exist with regard to this manuscript.
