# Supplementary material for: The TERT promoter mutation status and MGMT promoter methylation status, combined with dichotomized MRI‐derived and clinical features, predict adult primary glioblastoma survival
Source: Cancer Med. 2018 Jul 9;7(8):3704–12. doi: 10.1002/cam4.1666 (PMC6089138; doi:10.1002/cam4.1666)
Supplement: Supplementary file 1 [file CAM4-7-3704-s001.pdf]

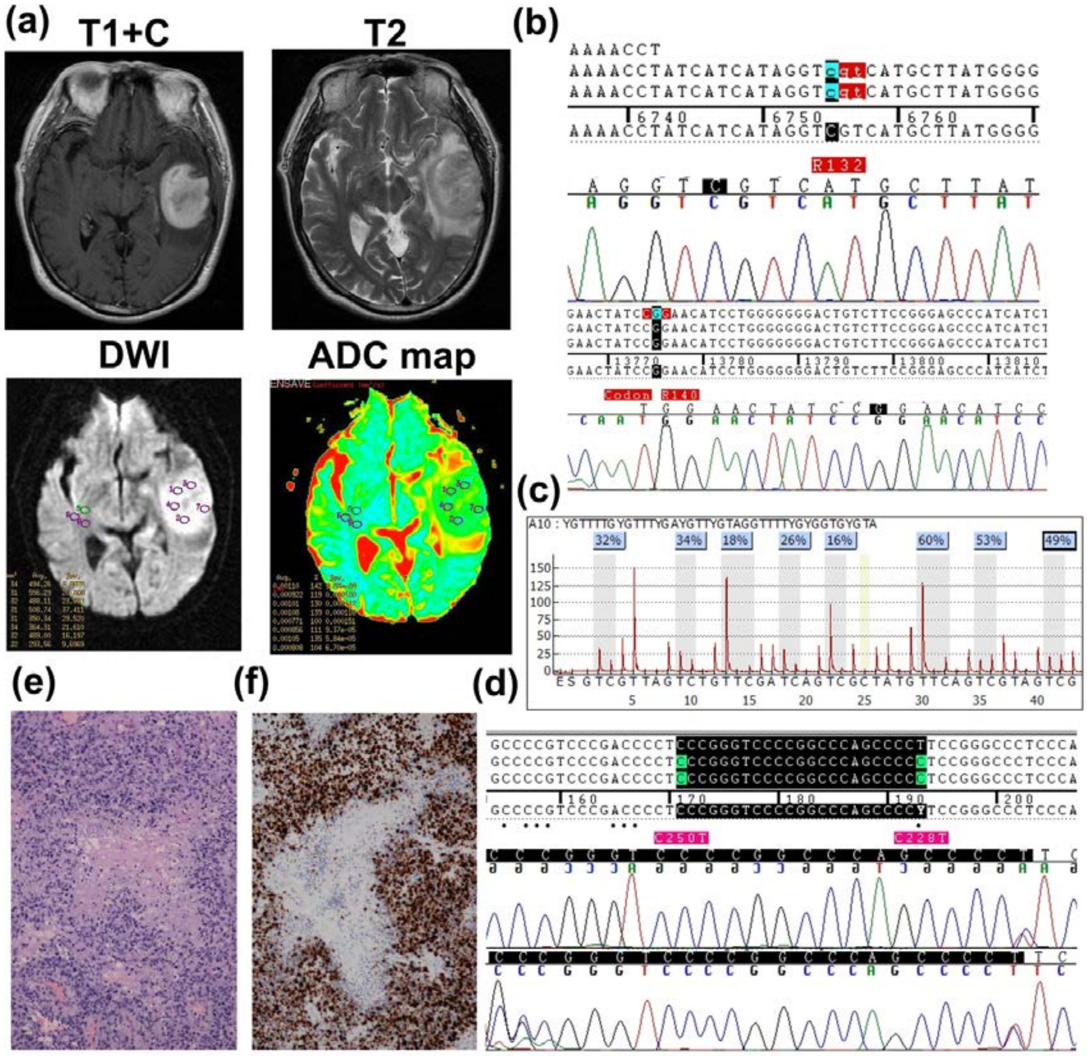

Supplemental Figure 1. Results obtained for a typical primary GBM patient. **(a)** MRI showing contrast-enhanced T1-weighted imaging, T2 imaging, DWI imaging and ADC map. **(b)** Sanger sequencing showing wild-type IDH1/2. **(c)** Pyrosequencing showed that the MGMT methylation frequency was 36%. **(d)** Sanger sequencing showed the mutation in the TERT promoter (C228T). **(e)** Histopathological HE staining showing GBM (WHO IV). **(f)** IHC staining for Ki-67 (high Ki-67 LI).

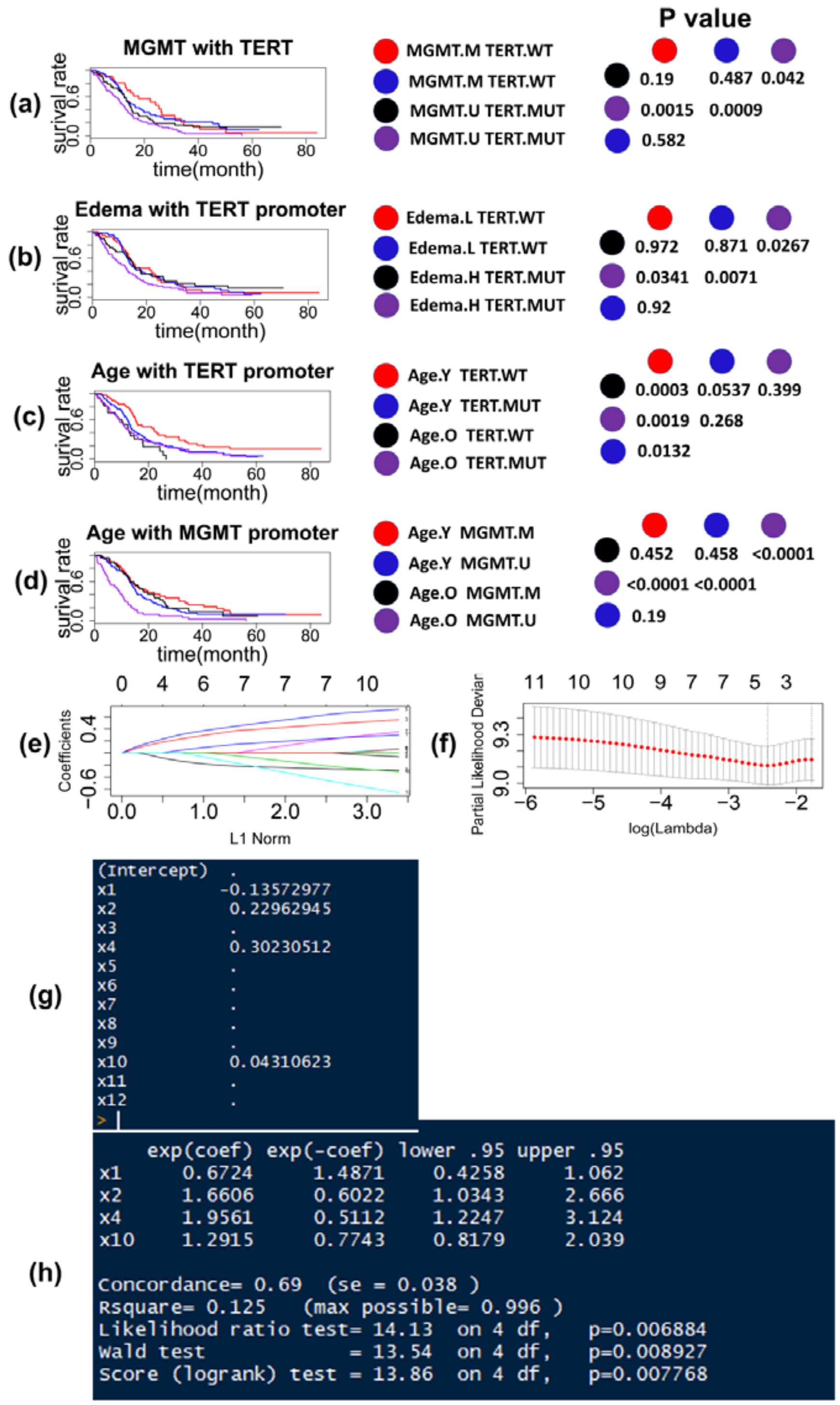

Supplemental Figure 2. (a)-(d) *P* values obtained from a log-rank test of the survival of the four subgroups in Figures 3 and 4. **(e)** LASSO coefficient profiles of the adult primary GBM-related factors in the subgroup with an unmethylated MGMT promoter and TERT promoter mutations. **(f)** Optimal lambda selection through cross-validation. **(g)** Nonzero coefficients in the LASSO analysis according to the cross-matching test. **(h)** Cox PH regression analysis for KPS, Edema, Age and Ki-67. In (g) and (h), x1 = KPS, x2 = edema, x3 = deep white matter invasion, x4 = age, x5 = ADC, x6 = T1/Flair ratio, x7 = necrosis, x8 = CET, x9 = cysts, x10 = Ki-67, x11 = gender, and x12 = nCET.
